# Temozolomide Enhances Triple-Negative Breast Cancer Virotherapy In Vitro

**DOI:** 10.3390/cancers10050144

**Published:** 2018-05-17

**Authors:** Rodolfo Garza-Morales, Roxana Gonzalez-Ramos, Akiko Chiba, Roberto Montes de Oca-Luna, Lacey R. McNally, Kelly M. McMasters, Jorge G. Gomez-Gutierrez

**Affiliations:** 1 The Hiram C. Polk Jr., MD, Department of Surgery, School of Medicine, University of Louisville, Louisville, KY 40202, USA; rod.ggarza@gmail.com (R.G.-M.); rgonzalezramos01@bellarmine.edu (R.G.-R.); mcmasters@louisville.edu (K.M.M.); 2 Department of Histology, School of Medicine, Autonomous University of Nuevo Leon, Monterrey 64460, NL, Mexico; rrrmontes@yahoo.com; 3 Department of Surgery, School of Medicine, Wake Forest University, Winston-Salem, NC 27109, USA; achiba@wakehealth.edu; 4 Department of Cancer Biology, Wake Forest Comprehensive Cancer Center, Wake Forest University, Winston-Salem, NC 27109, USA; lacey_mcnally@hotmail.com; 5 James Graham Brown Cancer Center, School of Medicine, University of Louisville, Louisville, KY 40202, USA

**Keywords:** oncolytic, adenovirus, triple-negative, breast cancer, temozolomide, autophagy, virotherapy

## Abstract

Triple-negative breast cancer (TNBC) is one of the most aggressive types of cancer, and treatment is limited to chemotherapy and radiation. Oncolytic virotherapy may be a promising approach to treat TNBC. However, oncolytic adenovirus (OAd)-based mono-therapeutic clinical trials have resulted in modest outcomes. The OAd potency could be increased by chemotherapy-induced autophagy, an intracellular degradation system that delivers cytoplasmic constituents to the lysosome. In this study, the ability of alkylating agent temozolomide (TMZ)-induced autophagy to increase OAd replication and oncolysis in TNBC cells was evaluated. Human TNBC MDA-MB-231 and HCC1937 cells and mouse 4T1 cells were infected with an OAd expressing the red fluorescent protein mCherry on the virus capsid (OAdmCherry) alone or in combination with TMZ. TNBC cells treated with OAdmCherry/TMZ displayed greater mCherry and adenovirus (Ad) early region 1A (E1A) expression and enhanced cancer-cell killing compared to OAdmCherry or TMZ alone. The combined therapy-mediated cell death was associated with virus replication and accumulation of the autophagy marker light chain 3 (LC3)-II. Overall, this study provides experimental evidence of TMZ’s ability to increase oncolytic virotherapy in both human and murine TNBC cells.

## 1. Introduction

Breast cancer is the most common malignancy in women and one of the three most common cancers worldwide [1]. Triple-negative breast cancer (TNBC) accounts for approximately 12–17% of all breast cancers and is more likely to affect younger women, African Americans, Hispanics, and/or those with breast cancer 1 (*BRCA1*) gene mutations. TNBC is a specific subtype of tumor that lacks the expression of estrogen receptors (ERs), progesterone receptors (PgRs), and human epidermal growth factor receptor type 2 (HER2). As a group, patients with TNBC have a relatively poor prognosis because of an inherently aggressive clinical behavior and a lack of molecular targets for therapy [2,3]. 

Cytotoxic chemotherapy is the primary treatment option for patients with TNBC in both early and advanced stages of the disease [3]. Studies of neoadjuvant chemotherapy with agents such as taxanes and anthracyclines have consistently reported high response rates, but despite optimal systemic chemotherapy, fewer than 30% of women with metastatic breast cancer survive longer than 5 years from diagnosis, and virtually all women with metastatic TNBC will ultimately die of their disease [4,5,6]. Therefore, an alternative approach that selectively targets cancer cells while sensitizing TNBC to chemotherapy must be developed. 

One of the most promising approaches for the treatment of malignant tumors is the use of oncolytic adenovirus (OAd). OAd’s are modified to replicate, spread, and induce oncolytic cell death in cancer cells but not in normal cells [7]. However, many clinical trials have revealed that monotherapy with OAd shows limited therapeutic effects, as its efficacy has been limited to oncolytic cell death [8]. For this reason, a combined therapy composed of OAd’s and other treatment modalities with different mechanisms of cell death is more likely to have success in the clinic. Several preclinical and clinical studies have demonstrated that OAd’s produce synergistic antitumor effects in combination with other treatment modalities, such as radiotherapy and chemotherapeutic agents [9,10,11,12,13].

Autophagy is the mechanism that involves cell degradation of unnecessary or dysfunctional cellular components. The breakdown of cellular components can ensure cellular survival during starvation and stress by maintaining cellular energy levels. Autophagy has a dual role, acting as a survival mechanism and as a caspase-independent form of programmed cell death [14,15]. We have shown that OAd induces autophagy in lung cancer cells and that autophagy inhibition with 3-methyladenine (3-MA), an autophagy inhibitor, decreases viral replication, whereas rapamycin, an autophagy inducer, increases OAd replication [16].

Temozolomide (TMZ) is a second-generation imidazotetrazine pro-drug that undergoes spontaneous conversion under physiological conditions to the active alkylating agent 5-(3-dimethyl-1-triazenyl) imidazole-4-carboxamide (MTIC) [17]. TMZ has been used for the treatment of a variety of malignancies, such as glioblastoma multiforme, astrocytoma, non-small cell lung carcinoma, melanoma, and breast cancer [18,19,20,21]. 

Several studies have demonstrated that TMZ-induced autophagy can enhance oncolytic virotherapy in melanoma and glioblastoma xenograft models [22,23,24,25]. We recently found that TMZ-induced autophagy enhanced OAd replication and oncolysis in human lung cancer cell lines and that the combination treatment led to a synergistic cancer-cell killing effect. Moreover, the combined therapy of OAd with TMZ resulted in superior lung tumor suppression in vivo over that of either treatment alone. Our data indicated that the enhanced anti-tumor activity was at least in part due to an OAd-mediated cytopathic effect (CPE), apoptosis, and autophagy induction [26]. More importantly, TMZ did not increase virus replication and oncolysis in human and mouse non-cancerous lung cells. This suggests that a combined therapy approach is safe for non-cancerous cells [27]. 

In the current study, we evaluated whether alkylating agent TMZ-induced autophagy enhances OAd replication and oncolysis in human and mouse TNBC cells as well as the cell death mechanisms of the combined treatment. Our results indicate that TMZ enhances OAd replication and oncolysis in TNBC cells. We also show that an increase in autophagy induction is associated with an increase in TNBC oncolytic cell death. 

## 2. Results

### 2.1. Evaluation of OAd-Mediated CPE and TMZ-Induced Cytotoxicity in Human TNBC Cells

**Figure 1:**
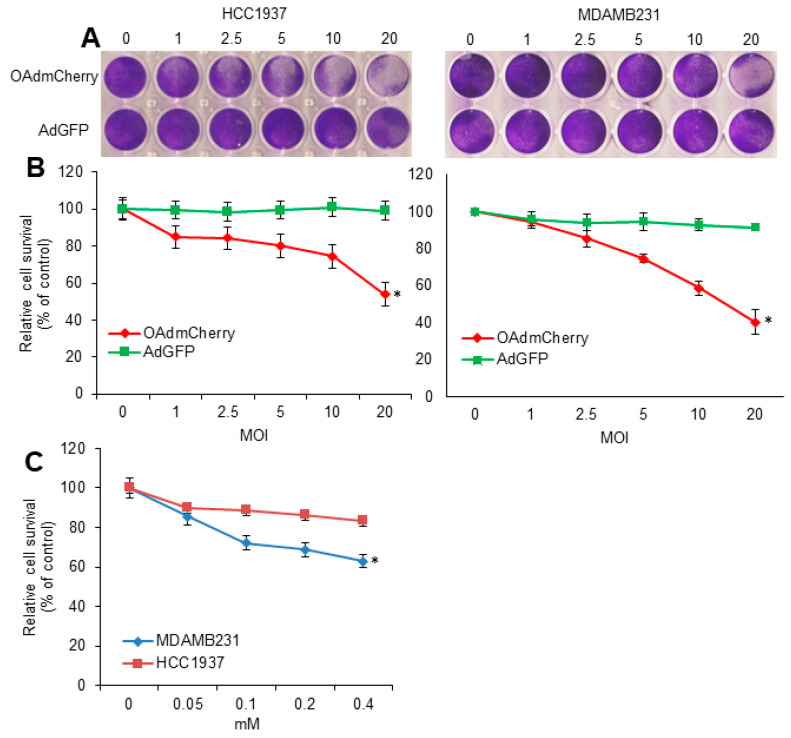
Oncolytic adenovirus expressing mCherry (OAdmCherry) and temozolomide (TMZ) have a cell-killing effect on human triple-negative breast cancer (TNBC) cells: (**A**) HCC1937 and MDA-MB-231 cells were infected with OAdmCherry or adenovirus expressing green fluorescent protein (AdGFP) at different multiplicity of infection concentrations for 72 h. Crystal violet staining was used to evaluate cytopathic effect (CPE). A representative staining of three independent experiments is shown. (**B**) Relative cell survival was calculated by measuring the absorbance of solubilized dye at 590 nm. (**C**) HCC1937 and MDA-MB-231 cells were treated with TMZ at different concentrations for 72 h. Cell survival was calculated by MTT assay. Results represent the mean of three repeated measurements ± standard deviation (SD; error bars) (* *p* < 0.05).

Human TNBC cell lines MDA-MB-231 and HCC1937 were infected with an OAd expressing mCherry (OAdmCherry) or a replication-deficient adenovirus expressing green fluorescent protein (AdGFP). At 72 h post infection, in both cell lines, crystal violet staining revealed that an OAdmCherry-mediated CPE increased in a virus-dose-dependent manner. In contrast, AdGFP-treated cells did not induce CPE, even at the highest multiplicity of infection (MOI) concentration of 20 (Figure 1A). Sensitivity to OAdmCherry was greater in MDA-MB-231 cells as compared to HCC1937 cells. For example, OAdmCherry at a MOI concentration of 10 induced 50% cell viability in MDA-MB-231, whereas 74% cell viability was observed in HCC1937 (Figure 1B). Next, TMZ-mediated cytotoxicity was determined. Human TNBC cell lines were treated at increasing TMZ concentrations. At 72 h post treatment, a 3-(4,5-dimethylthiazol-2-yl)-2,5-diphenyltetrazolium (MTT) assay revealed that cell viability decreased in a dose-dependent manner. HCC1937 cells displayed greater resistance to TMZ than MDA-MB-231. A TMZ dose of 0.4 mM resulted in 20% cell viability in HCC1937 cells and 40% cell viability in MDA-MB-231 cells (Figure 1C). 

### 2.2. TMZ Increases Viral Infection and Ad E1A Gene Expression in Human TNBC Cells

**Figure 2:**
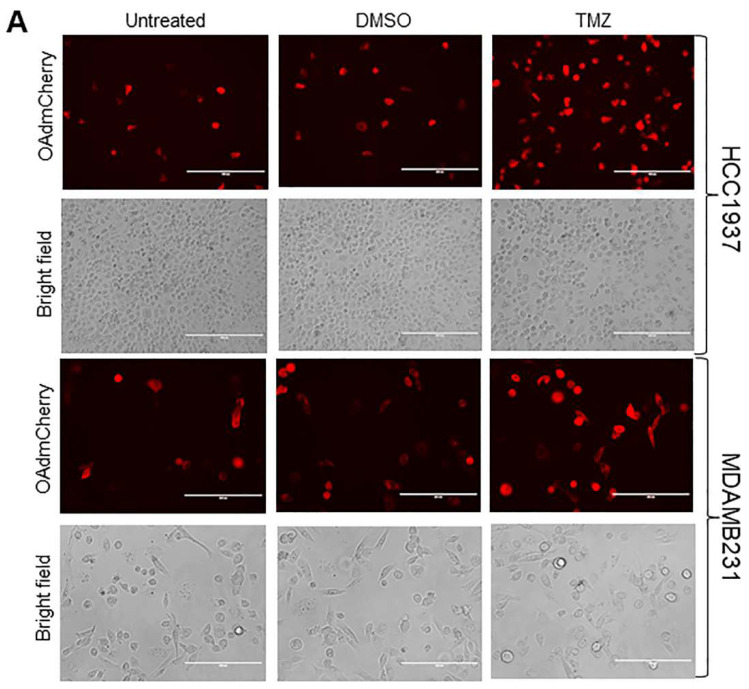

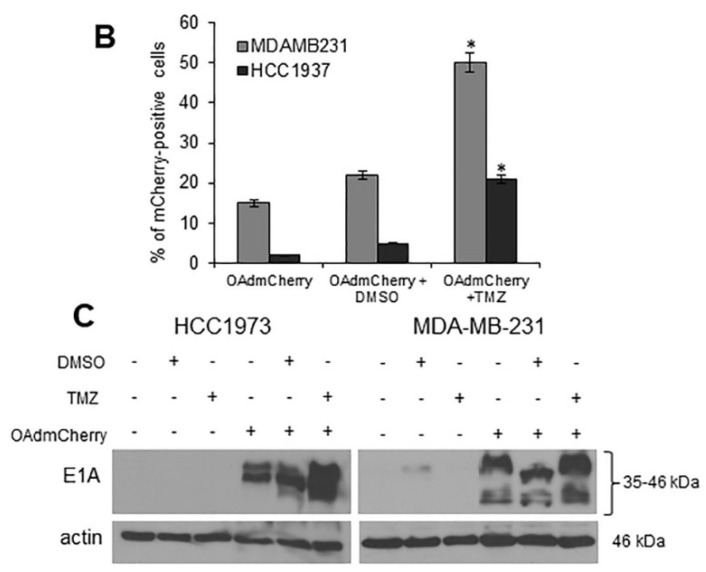
Effect of temozolomide (TMZ) treatment on virus infection and adenovirus early region 1A (E1A) expression in human triple-negative breast cancer (TNBC) cells: (**A**) Human TNBC cells were infected with oncolytic adenovirus mCherry (OAdmCherry) at a multiplicity of infection concentration of 2.5 alone or in combination with TMZ or vehicle dimethyl sulfoxide (DMSO). Expression of mCherry was evaluated by fluorescence microscopy. Scale: 200 µm. (**B**) Percentage of mCherry-positive cells calculated relative to number of cells in the field. Results represent the mean of three repeated measurements ± standard deviation (SD; error bars) (* *p* < 0.05). (**C**) Expression of Ad E1A was evaluated by Western blot assay at 24 h post treatment. Actin was used as a loading control. A representative assay is shown from three performed.

Human TNBC cell lines were infected with OAdmCherry alone or in combination with TMZ or a vehicle control dimethyl sulfoxide (DMSO). At 24 h post infection, mCherry expression was visualized by fluorescence microscopy (Figure 2A). OAdmCherry-infected HCC1937 and MDA-MB-231 cells displayed 2% and 15% mCherry-positive cells, respectively. Treatment with DMSO slightly increased mCherry expression to 5% and 22%, respectively. In contrast, a greater mCherry expression was observed in OAdmCherry/TMZ-treated cells, increasing to 21% and 50%, respectively (Figure 2B). These results suggest that TMZ increases OAdmCherry infection as early as 24 h post treatment with TMZ. To further validate adenovirus infection, the expression of Ad E1A, a key component of Ad replication machinery, was evaluated by Western blot assay. Similarly to the results observed for mCherry expression, Ad E1A expression levels were modest in cells infected with OAdmCherry alone or in combination with DMSO, whereas OAdmCherry/TMZ-treated cells exhibited greater levels of adenovirus (Ad) early region 1A (E1A) expression (Figure 2C). These results suggest that TMZ has the ability to increase OAd infection and Ad E1A expression in TNBC cells.

### 2.3. TMZ Facilitates Adenovirus Entry into Human TNBC Cells

**Figure 3:**
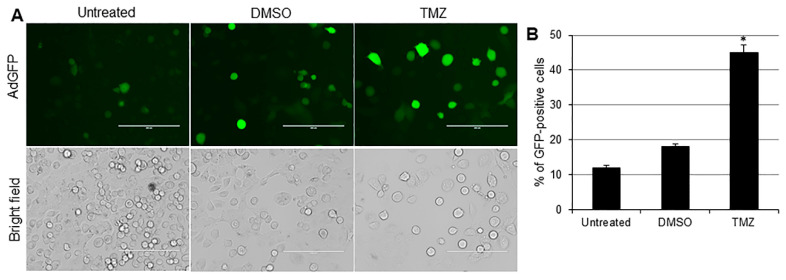
Temozolomide (TMZ) facilitates adenovirus entry into triple-negative breast cancer (TNBC) cells: (**A**) HCC1937 cells were infected with adenovirus expressing green fluorescent protein (AdGFP) at a multiplicity of infection concentration of 5 alone or in combination with TMZ (0.4 mM) or vehicle dimethyl sulfoxide (DMSO). Expression of GFP was evaluated by fluorescence microscopy. Scale: 200 µm. (**B**) Percentage of GFP-positive cells calculated relative to number of cells in the field. Results represent the mean of three repeated measurements ± standard deviation (SD; error bars) (* *p* < 0.05).

To further validate TMZ’s ability to facilitate the adenovirus entry into TNBC cells, the HCC1937 cell line was infected with an AdGFP alone or in combination with DMSO (drug vehicle control) or TMZ. At 24 h post infection, GFP expression was visualized by fluorescence microscopy (Figure 3A). AdGFP-infected HCC1937 cells displayed 12% GFP-positive cells. Treatment with DMSO slightly increased GFP expression to 18%, whereas a greater GFP expression was observed in TMZ-treated cells, increasing to 45% (Figure 3B). These results confirm that TMZ could facilitate adenovirus entry into TNBC cells. This result suggests that TMZ may represent a useful chemotherapeutic drug to increase adenovirus infection in those cancer cells that exhibit poor infectability. 

### 2.4. TMZ Enhances OAd-Mediated Oncolytic Cell Death Partly as a Result of Increased Virus Replication

**Figure 4:**
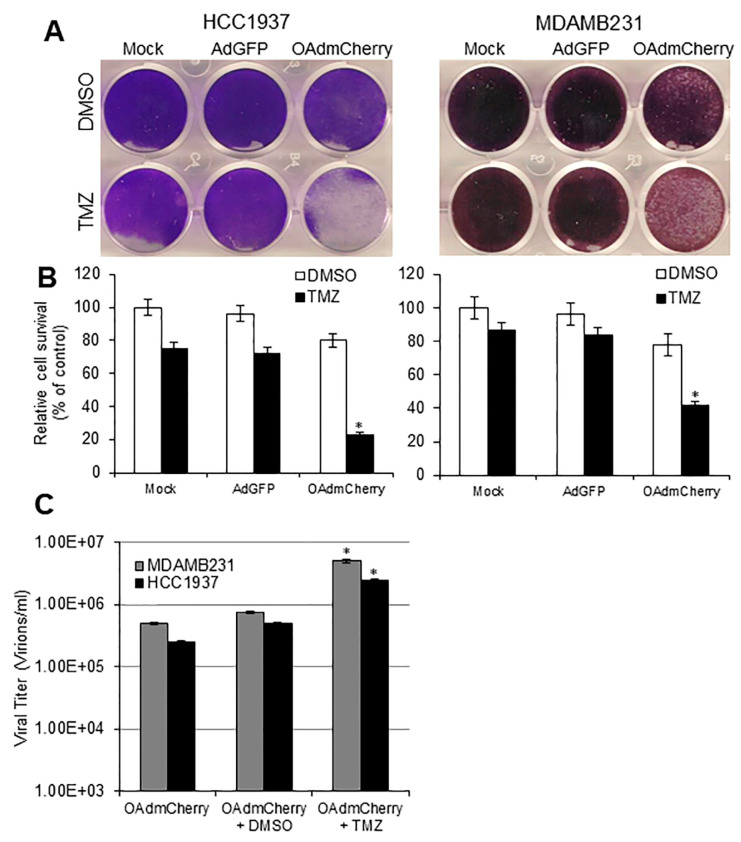
Temozolomide (TMZ) enhances oncolytic adenovirus (OAd)-mediated cytopathic effect (CPE) through increased viral replication: (**A**) Human triple-negative breast cancer (TNBC) cells were infected with OAdmCherry or adenovirus expressing green fluorescent protein (AdGFP) at a multiplicity of infection concentration of 2.5 alone or in combination with either dimethyl sulfoxide (DMSO) or TMZ. At 72 h post infection, crystal violet staining was used to evaluate CPE. A representative staining is shown of three experiments performed. (**B**) OAd-mediated CPE was calculated by measuring the absorbance of solubilized dye at 590 nm. Results represent the mean of three repeated measurements ± standard deviation (SD; error bars) (* *p* < 0.05). (**C**) Supernatants were collected and used to determine adenovirus yield from each cell line. Results represent the mean of three repeated measurements ± standard deviation (SD; error bars) (* *p* < 0.05).

Human TNBC cell lines were infected with either AdGFP or OAdmCherry as described above alone or in combination with TMZ or vehicle control DMSO. At 72 h post treatment, crystal violet staining (Figure 3A) showed that the combination of OAdmCherry and TMZ induced greater CPE in both HCC1937 and MDA-MB-231 cell lines (23% and 42% cell viability) as compared with either OAdmCherry alone (80% and 78% cell viability) or TMZ alone (75% and 87% cell viability) (Figure 4B). AdGFP did not induce cytotoxicity alone or in combination with TMZ (Figure 4A,B). TMZ increased OAdmCherry virus production approximately 10-fold in both cell lines compared to OAdmCherry-infected cells treated with vehicle control DMSO (Figure 4C). Overall, this suggests that TMZ increases OAdmCherry-mediated CPE in human TNBC cells via productive virus replication. 

### 2.5. Combination of OAdmCherry with TMZ Alters Autophagy in Human TNBC Cells

**Figure 5:**
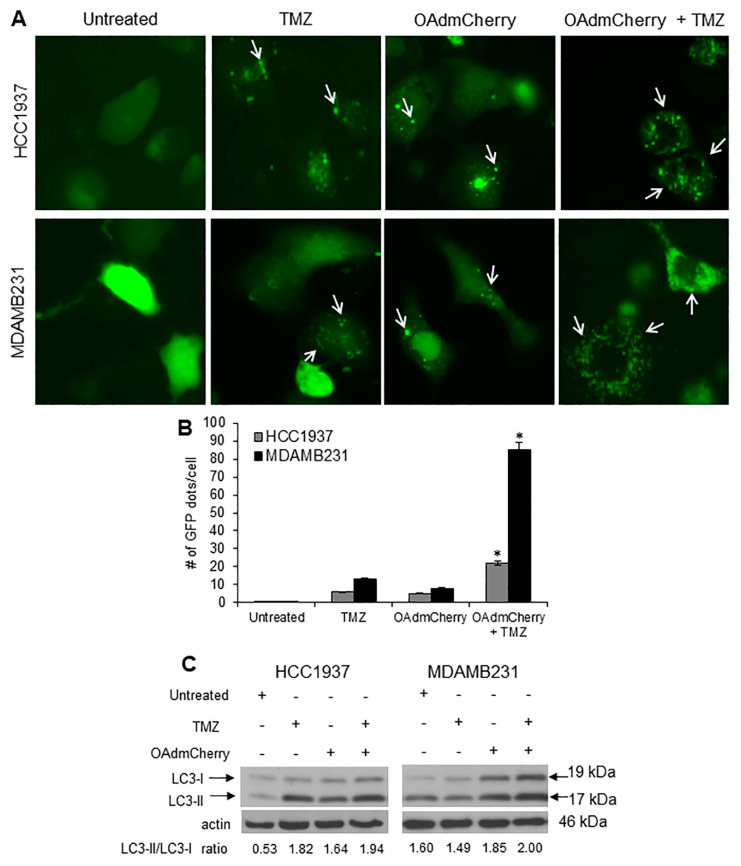
Combined therapy increases autophagosome formation and light chain 3 (LC3)-II accumulation: (**A**) Human triple-negative breast cancer (TNBC) cells were transfected with pEGFP-LC3 followed by treatment with temozolomide (TMZ) at 0.4 mM or oncolytic adenovirus mCherry (OAdmCherry) at a multiplicity of infection concentration of 10 alone or in combination. Integration of GFP-LC3 into the autophagosome is depicted by punctate structures (arrows) and was analyzed by fluorescence microscopy at 48 h post treatment. Images were taken at 40× magnification with the EVOS FL Imaging System (Advanced Microscopy Group) under 357/44 and 447/60 nanometers (nm) excitation and emission visualization, respectively. (**B**) Comparison of number of GFP dots per cell in untreated cells or cells treated with TMZ, OAdmCherry, or a combination of both. A representative experiment is shown from three performed (* *p* < 0.05). (**C**) Whole cell protein lysates were collected 24 h post treatment. Expression of LC3-I and LC3-II was detected by Western blot analysis; actin was used as a loading control. A representative experiment is shown from three performed.

Because TMZ is known to induce autophagy, we next investigated whether the combination of OAdmCherry with TMZ could enhance autophagy induction over that of either agent independently. HCC1937 and MDA-MB-231 cell lines were transfected with plasmid Enhanced Green Fluorescent Protein-Microtubule-associated protein 1A/1B-light chain 3 (pEGFP-LC3) followed by either TMZ or OAdmCherry alone or in combination. The formation of cytoplasmic punctate GFP fluorescence was then observed. The conversion of cytoplasmic diffuse GFP-LC3-1 to membrane-associated GFP-LC3-II formed punctate patterns, indicating LC3-II incorporation into the autophagosomes. This formation of punctate was observed 48 h after treatment (Figure 5A). TMZ-treated HCC1937 and MDA-MB-231 cells displayed an accumulation of the fluorescent punctate pattern. HCC1937 cells showed 6 dots per cell and MDA-MB-231 showed 13 dots per cell on average. OAdmCherry-infected HCC1937 and MDA-MB-231 cells displayed a similar effect; these cells showed five and eight dots per cell on average, respectively. Greater fluorescent punctate pattern accumulation was observed with the combined treatment. HCC1937 cells showed 22 dots per cell and MDA-MB-231 cells showed 85 dots per cell on average (Figure 5B). Next, the conversion of LC3-I to LC3-II, an autophagy marker [28], was evaluated. Western blot analysis revealed two reactive LC3 species: an upper band corresponding to LC3-I (19 kDa) and a lower band corresponding to LC3-II (17 kDa). In both TNBC cell lines, a marked accumulation of LC3-II was observed with the combined treatment as compared to untreated and TMZ- or OAdmCherry-treated cells (Figure 5C). These results suggest that the combination of OAdmCherry and TMZ alters autophagy in TNBC cell lines more so than either treatment alone. 

### 2.6. TMZ Increases OAdmCherry Infectivity and Ad E1A Expression, and Combined Therapy Strongly Inhibits Clonogenic Survival in Mouse TNBC Cells

**Figure 6:**
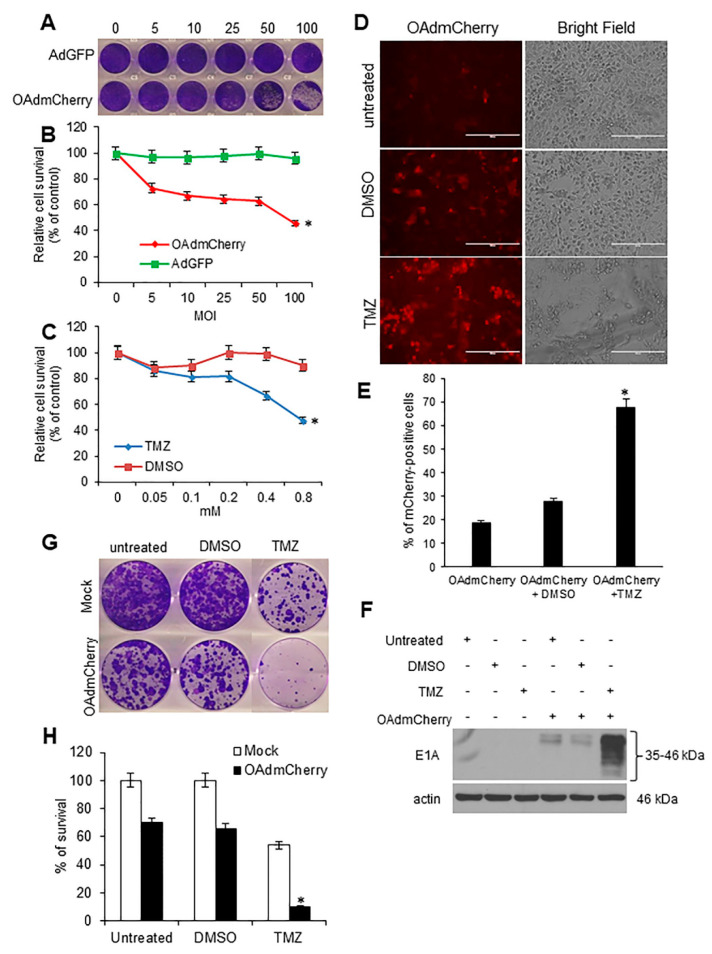
Combined therapy increases viral replication and strongly inhibits clonogenic survival in mouse triple-negative breast cancer (TNBC) cells: (**A**) 4T1 cells were infected with oncolytic adenovirus mCherry (OAdmCherry) or adenovirus expressing green fluorescent protein (AdGFP) at different multiplicity of infection (MOI) concentrations for 72 h. Crystal violet staining was used to evaluate cytopathic effect (CPE). A representative staining of three independent experiments is shown. (**B**) Relative cell survival was calculated by measuring the absorbance of solubilized dye at 590 nm. (**C**) 4T1 cells were treated with TMZ at different concentrations for 72 h. Cell survival was calculated by MTT assay. Results represent the mean of three repeated measurements ± standard deviation (SD; error bars) (* *p* < 0.05). (**D**) 4T1 cells were infected with OAdmCherry at a MOI concentration of 10 alone or in combination with TMZ or vehicle dimethyl sulfoxide (DMSO). Expression of mCherry was evaluated by fluorescence microscopy. Scale: 200 µm. (**E**) Percentage of mCherry-positive cells calculated relative to number of cells in the field. Results represent the mean of three repeated measurements ± standard deviation (SD; error bars) (* *p* < 0.05). (**F**) Expression of (Ad) E1A was evaluated 72 h post treatment by Western blot assay. Actin was used as a loading control. A representative assay is shown from three performed. (**G**) 4T1 cells were infected with OAdmCherry alone or in combination with TMZ or vehicle DMSO. Crystal violet staining was used to evaluate clonogenic survival. (**H**) Percentage of cell survival calculated relative to the untreated control. Results represent the mean of three repeated measurements ± standard deviation (SD; error bars) (* *p* < 0.05).

Because the mouse TNBC 4T1 cell line represents an animal stage IV human breast cancer, we further investigated whether the combined therapy of OAd with TMZ could be effective in this cell line. Previously we found that TMZ treatment facilitated AdGFP entry into 4T1 cells [27]. We first evaluated OAdmCherry-mediated CPE and TMZ-induced cytotoxicity in 4T1 cells. Mouse TNBC 4T1 cells were infected with OAdmCherry or AdGFP at increasing concentrations of MOI. At 72 h post infection, crystal violet staining revealed that OAdmCherry-mediated CPE increased in a virus-dose-dependent manner. In contrast, AdGFP-treated cells did not induce CPE even at the highest MOI concentration of 100 (Figure 6A). The 4T1 cells displayed greater resistance to OAdmCherry-mediated CPE as compared with human TNBC cell lines (Figure 1A). However, 4T1 cells responded relatively efficiently to OAdmCherry-mediated CPE. For example, OAdmCherry at a MOI concentration of 10 reduced cell viability to 66% (Figure 6B). Next, TMZ-mediated cytotoxicity was determined. 4T1 cells were treated at increasing TMZ concentrations. At 72 h post treatment, a MTT assay revealed that cell viability decreased in a dose-dependent manner. TMZ at 0.4 mM decreased cell viability by 67% in 4T1 cells (Figure 6C). We then investigated whether TMZ enhances viral replication and spread. The 4T1 cells were infected with OAdmCherry alone or in combination with TMZ or DMSO. At 72 h post infection, mCherry expression was visualized by fluorescence microscopy (Figure 5D). OAdmCherry-infected 4T1 cells displayed 20% mCherry-positive cells. Treatment with DMSO slightly increased mCherry expression to 25%. In contrast, the combined treatment with TMZ significantly increased mCherry expression to 65% (Figure 5E). To further assess the effect of TMZ upon adenovirus (Ad) replication, Ad E1A protein expression was evaluated by Western blot assay. Similarly to the results observed for mCherry expression, Ad E1A expression levels were modest in both OAdmCherry alone or in combination with DMSO, whereas TMZ-treated cells exhibited greater levels of Ad E1A expression (Figure 5F). These results suggest that TMZ has the ability to increase OAd replication in murine 4T1 TNBC cells. Finally, the therapeutic effect of the combination of OAdmCherry with TMZ was evaluated in vitro; single colonies were visualized by crystal violet staining (Figure 5G). The clonogenic survival assay revealed that in 4T1 cells, TMZ-alone and OAdmCherry-alone induced 45% and 65% survival, respectively, whereas the combined therapy (OAdmCherry/TMZ) resulted in only 10% survival (Figure 5H). These data also suggest that the combination of OAdmCherry and TMZ has a potent inhibitory effect upon colony formation in murine TNBC cells.

## 3. Discussion

Oncolytic virotherapy is an emerging treatment modality for the treatment of advanced solid tumors refractory to current therapies. However, OAd’s used as monotherapy have shown limited therapeutic effects because their efficacy has been limited to oncolytic cell death. Therefore, combining oncolytic Ad’s with other treatment is mandatory to achieve their full potential. In the present study, using the human TNBC cell lines HCC1937 and MDA-MB-231 and murine TNBC cell line 4T1, we demonstrated that combining OAdmCherry with the alkylating agent TMZ led to an enhanced OAd infection and oncolysis, which was associated with increased autophagosome formation and accumulation of LC3-II. The mechanism of TMZ-induced increase in Ad replication is not elucidated in the current study, and further research is necessary to determine the role of autophagy in the observed increased Ad replication and oncolysis.

TNBC is one of the most aggressive and complicated types of breast cancer to treat. Chemotherapy remains to this day the standard therapeutic approach for TNBC at all stages. However, many tumors are highly resistant to chemotherapy, causing patients to relapse quickly after treatment; this is mostly because of genomic and chromosomal instability present in TNBC. Therefore, a combination therapy involving several treatment modalities that can surpass this difficulty and reduce the dose of chemotherapeutic drugs while maintaining therapeutic efficacy is more likely to have success in the clinic [29].

There have been various advances to enhance oncolytic virotherapy using different chemotherapeutic drugs, radiotherapy, and immune-checkpoint inhibitors [30]. A wide range of clinical and pre-clinical studies have shown that combining different treatment modalities has resulted in in vitro and in vivo synergies through various mechanisms of cell death. Synergy has been recorded between the alkylating agent TMZ and OAd in various types of cancer, such as in advanced glioma [31,32,33,34]. 

Previously, it was reported that the MDA-MB-436 human TNBC cell line, when treated with a triple combination of TMZ; 4-hydroperoxycyclophosphamide (4-HPCP); an active metabolite of the prodrug cyclophosphamide (CP); and Ad5/3-D24-GMCSF, a 5/3-capsid chimeric OAd coding for GM-CSF, had increased immunogenic cell killing and autophagy [31]. In another study, it was reported that CP at low doses increased the efficacy of Ad5/3-DM4-GMCSF in MDA-MB-436 cells, and similar effects were observed in an orthotopic TNBC xenograft mouse model [35].

Autophagy plays a critical role on OAd-mediated CPE in cancer cells. The basic mechanism of autophagy involves cell degradation of unnecessary or dysfunctional cellular components. Autophagy has dual roles, acting as a survival mechanism and as a caspase-independent form of programmed cell death [14]. Previously, it was reported that OAd induces autophagy by increasing the conversion of LC3-I to LC3-II. Additionally, the inhibition of autophagy with 3-MA resulted in a decreased expression of adenoviral proteins and viral replication, and the induction of autophagy with rapamycin increased OAd replication [16]. In contrast, other studies have shown that deletion of 24 amino acids in the *E1A* gene and with the tripeptide Arg-Gly-Asp (Delta-24-RGD), an OAd whose infectivity in cancer cells is enhanced through the insertion of a RGD-4C motif in the high affinity (HI) loop of the adenoviral fiber protein, and OBP-405, an OAd regulated by the human telomerase reverse transcriptase promoter (hTERT-Ad, OBP-301) with a tropism modification (RGD), induced the autophagic cell death of glioblastoma cells, whereas autophagy inhibitors did not affect the replication of Delta-24-RGD and OBP-405 [36,37].

We recently found that TMZ is able to render murine and human lung cancer cells susceptible to oncolytic virotherapy. The enhanced killing effect induced by TMZ and Adhz60 (*E1b* deleted Ad serotype 5) was due to productive virus replication and autophagy induction [26,27]. In addition, the OAd used in the previous studies was demonstrated to be safe for normal human and mouse cell lines. In this study, we confirmed that TMZ treatment sensitizes murine cancer cells, particularly 4T1 TNBC cells, and represents an animal stage IV human breast cancer model. Therefore, this syngeneic mouse model has significant clinical relevance because it mimics the clinical situation of TNBC patients.

In conclusion, this study provides the experimental evidence showing that TMZ can be used to enhance oncolytic virotherapy in TNBC cells, which may represent an alternative approach to destroy TNBC tumors in patients with resistance to chemotherapy. Most importantly, human TNBC cells were efficiently destroyed by the combined therapy of OAd with TMZ. In addition, these chemovirotherapies may allow for the use of less-toxic doses to achieve therapeutic efficacy and prime the immune system to reduce the chances of cancer recurrences. 

## 4. Materials and Methods 

### 4.1. Cell Lines and Culture Conditions

Human embryonic kidney cell line (HEK-293) (Cat# CRL-1573), human TNBC HCC1937 (Cat# CRL-2336) and MDA-MB-231 cells (Cat # HTB-26), and murine TNBC 4T1 cells (Cat# CRL-2539) were purchased from the American Type Culture Collection (ATCC) (Manassas, VA, USA). HCC1937 and 4T1 cells were grown in RPMI-1640 medium (Cat# 10-040-CV). MDA-MB-231 and HEK-293 cells were grown in Dulbecco’s Modified Eagle’s Medium (DMEM) (Cat# 10-013-CV). All media were supplemented as previously described [38]. All cell culture reagents were obtained from Corning Cellgro (Manassas, VA, USA).

### 4.2. Adenoviral Vectors and Drugs

A replication-deficient adenoviral vector expressing green fluorescent protein (AdGFP) under regulation of a cytomegalovirus (CMV) promoter was used as a negative control for virus replication as previously described [38]. The conditionally replicating adenovirus expressing mCherry red fluorescent protein on the capsid was constructed by homologous recombination in *Escherichia coli* (BJ5183 strain) using the fiber gene modified AdEasy-1 backbone vector AdEz-F5/3 (Ad5Δ*E1*/Δ*E3*-F5/3) and a modified pShuttle vector pSlΔ24-pIX-mCherry. This shuttle vector contained the mCherry coding sequence inserted downstream from the Ad5 minor capsid pIX gene to generate a C-terminal pIX fusion and a 24-basepair deletion in the Ad5 E1A gene coding sequence (Δ24) [39]. TMZ stock solution of 50 mM was prepared in DMSO and stored at −20 °C. The final volume of TMZ and vehicle control DMSO added to the cell cultures was less than 1%. All drugs were purchased from Sigma-Aldrich (St. Louis, MO, USA).

### 4.3. Single and Combined Therapies

A total of 2.5 × 10^4^ cells were plated in a 24-well plate and treated 24 h later with the indicated therapy. Viral infection was performed at an indicated MOI concentration, whereas TMZ treatment was performed at an indicated millimolar (mM) concentration. OAdmCherry-mediated CPE was evaluated at 72 h post infection by crystal violet staining. Suspended cells were removed by aspiration; the remaining adherent cells were then fixed with 3.7% formaldehyde for 3 min at room temperature. The excess formaldehyde was washed with Phosphate-buffered saline (PBS); the cells were then stained using 1% crystal violet at room temperature for 3 min. Excess crystal violet was washed away with PBS. Plates were then scanned using an HP Scanjet 4070 scanner (HP, Palo Alto, CA, USA). The remaining crystal violet was then solubilized with a 2% sodium dodecyl sulfate (SDS) solution, and the sample absorbances were measured at 590 nm using a Synergy HT Multi-Mode Microplate Reader (Bio-Tek, Winooski, VT, USA). The absorbance (OD) values of each treatment were then normalized to mock-treated cells converting each sample OD into the cell viability percentage (%) according to the following formula: cell viability % = (OD of treated cells/OD of mock-treated cells) × 100%, as described previously [40]. The expression of mCherry was assessed 24 h after OAd infection using a Leica DM1000 fluorescence microscope with an N2.1 filter at 587 and 610 nm for excitation and emission, respectively. 

Cell viability was assessed 72 h after TMZ treatment by measuring the conversion of tetrazolium salt 3-(4,5-dimethylthiazol-2-yl)-2,5-diphenyltetrazolium (MTT) to formazan, as described previously [38]. The supernatant from each plate was collected for measurement of absorbance at a wavelength of 570 nm. The results are expressed as the percentage of live cells. For control infection, we used cell-line-specific media alone without virus or DMSO instead of TMZ. For combined therapies, cells were treated with OAdmCherry or AdGFP at a MOI concentration of 2.5 or TMZ of 0.4 mM, respectively.

### 4.4. Adenovirus Titer Assay

Cells were infected with OAdmCherry alone or were treated as described in the previous section; 72 h after treatment, supernatants were collected and centrifuged for 10 min at 14,000 rpm. The supernatants were then transferred to a new tube to eliminate cell debris and/or cells in suspension that may have contained Ad’s. Supernatants were diluted serially by using the median tissue culture infective dose, the amount of a pathogenic agent that would produce pathological change in 50% of cell cultures inoculated (TCID50), or by using the end-point dilution method with HEK-293 cells seeded on 96-well plates. Briefly, HEK 293 cells were seeded in 96-well plates at a density of 10^3^ (cells per well) and were then infected with 10-fold serially diluted viruses. CPE was recorded and scored after incubation for 7 days. The reduction percentage in virus titer was calculated by the following formula: reduction % =  [(titer of control group − titer of experimental group)/titer of control group] × 100% [16,41].

### 4.5. Western Blot Analysis

Cells were harvested and lysed with radioimmunoprecipitation assay (RIPA) buffer, as described previously [42]. Cell lysates were centrifuged, and the protein concentration was determined by a Pierce bicinchoninic acid assay (BCA) protein kit (Thermo Scientific, Waltham, MA, USA). Equal amounts of cellular protein were electrophoresed on 10–12% SDS–polyacrylamide gels and transferred to Hybond-Polyvinylidene fluoride (PVDF) () membranes (GE Healthcare Life Sciences, Pittsburgh, PA, USA). The primary antibodies used were rabbit anti-LC3 polyclonal antibody (Sigma-Aldrich, St. Louis, MO, USA), mouse anti-adenovirus type 5 E1A (BD Pharmingen, San Diego, CA, USA), and rabbit anti-human actin (Sigma-Aldrich, St. Louis, MO, USA). Next, the membranes were incubated with anti-mouse immunoglobulin (Ig) or anti-rabbit Ig, peroxidase-linked, species-specific whole antibody (Thermo Fisher Scientific, Waltham, MA, USA). (Electrochemiluminescence) (ECL) reagents were used to detect the signals according to the manufacturer’s instructions (GE Healthcare Life Sciences, Pittsburgh, PA, USA). The scanned band intensities were quantified using Gel-pro Analyzer 4.0 software (Media Cybernetics, Rockville, MD, USA) according to the manufacturer’s instructions. Densitometric values for each band were expressed as integrated optical density (I.O.D.) and were normalized to actin expression. 

### 4.6. GFP-LC3 Puncta

Plasmid vector containing green fluorescent protein linked to microtubule-associated protein 1 LC3 was used to detect autophagosome formation in TNBC cell lines [28]. At 24 h post transfection, cells were treated with TMZ at 0.4 mM or OAdmCherry at a MOI concentration of 2.5 alone or in combination. At 48 h post treatment, cells were examined under a fluorescence microscope. Cells were classified as having a predominantly diffuse GFP stain or having numerous punctate structures representing autophagosomes. Images were taken at 40x magnification with the EVOS FL Imaging System (Thermo Fisher Scientific, Waltham, MA, USA) under 357/44 and 447/60 nanometers (nm) excitation and emission visualization, respectively. The percentage of cells with GFP puncta were calculated as the proportion of cells with GFP puncta divided by the total number of GFP expressing cells.

### 4.7. Clonogenic Survival Assay

A clonogenic survival assay was performed according to a previous publication [43]. Mouse 4T1 TNBC cells were treated with DMSO, TMZ, or OAdmCherry alone or in combination at indicated concentrations; 24 h post treatment, the cells were trypsinized and plated at a cell density of 1 × 10^3^ per well in a 6-well plate. The cells were cultured for 10 days, fixed with 3.7% paraformaldehyde, and stained with 1% crystal violet. Colonies with ≥50 cells were counted. The plating efficiency was calculated for each condition, with the surviving fraction calculated relative to the untreated control, as described previously [43,44,45].

### 4.8. Statistical Analysis

One- and two-way ANOVA was used to determine differences in cell viability across different treatments. Statistical differences between combined treatments (TMZ/OAdmCherry) and either agent alone were determined by the significance of the interaction effect of the dose and virus. Differences in cell viability across combination therapies were analyzed by one-way ANOVA. Post hoc testing was performed with Tukey’s adjustment to control for a significance level of 0.05.
