# Correction: Garza-Morales et al. Temozolomide Enhances Triple-Negative Breast Cancer Virotherapy In Vitro. *Cancers* 2018, *10*, 144

**DOI:** 10.3390/cancers16071432

**Published:** 2024-04-07

**Authors:** Rodolfo Garza-Morales, Roxana Gonzalez-Ramos, Akiko Chiba, Roberto Montes de Oca-Luna, Lacey R. McNally, Kelly M. McMasters, Jorge G. Gomez-Gutierrez

**Affiliations:** 1 The Hiram C. Polk Jr., MD, Department of Surgery, School of Medicine, University of Louisville, Louisville, KY 40202, USA; rod.ggarza@gmail.com (R.G.-M.); rgonzalezramos01@bellarmine.edu (R.G.-R.); mcmasters@louisville.edu (K.M.M.); 2 Department of Histology, School of Medicine, Autonomous University of Nuevo Leon, Monterrey 64460, NL, Mexico; rrrmontes@yahoo.com; 3 Department of Surgery, School of Medicine, Wake Forest University, Winston-Salem, NC 27109, USA; achiba@wakehealth.edu; 4 Department of Cancer Biology, Wake Forest Comprehensive Cancer Center, Wake Forest University, Winston-Salem, NC 27109, USA; lacey_mcnally@hotmail.com; 5 James Graham Brown Cancer Center, School of Medicine, University of Louisville, Louisville, KY 40202, USA

## Error in Figure

In the original publication [1], there was a mistake in the right side of Figure 1A. The image of a crystal violet plate of MDA-MB-231 cells was inadvertently duplicated from a previous manuscript (reference [27] in original publication [1]). The corrected Figure 1A and its quantification have been updated in the figure below. A new set of experiments was performed to replace the duplicated crystal violet plate and generate a new quantification graph. The authors apologize for any inconvenience caused and state that the scientific conclusions remained unaffected. This correction was approved by the Academic Editor. The original publication has also been updated.

## Figures and Tables

**Figure 1:**
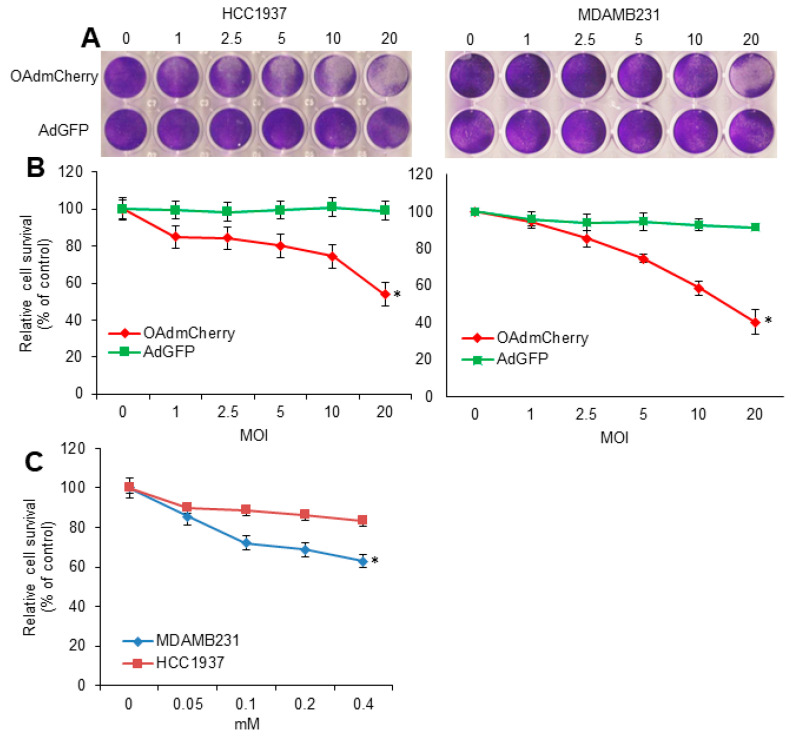
Oncolytic adenovirus expressing mCherry (OAdmCherry) and temozolomide (TMZ) have a cell-killing effect on human triple-negative breast cancer (TNBC) cells: (**A**) HCC1937 and MDA-MB-231 cells were infected with OAdmCherry or adenovirus expressing green fluorescent protein (AdGFP) at different multiplicity of infection concentrations for 72 h. Crystal violet staining was used to evaluate cytopathic effect (CPE). A representative staining of three independent experiments is shown. (**B**) Relative cell survival was calculated by measuring the absorbance of solubilized dye at 590 nm. (**C**) HCC1937 and MDA-MB-231 cells were treated with TMZ at different concentrations for 72 h. Cell survival was calculated by MTT assay. Results represent the mean of three repeated measurements ± standard deviation (SD; error bars) (* *p* < 0.05).

## References

[1] Garza-Morales R., Gonza-lez-Ramos R., Chiba A., de Oca-Luna R.M., McNally L.R., McMasters K.M., Gomez-Gutierrez J.G. (2018). Temozolomide Enhances Triple-Negative Breast Cancer Virotherapy In Vitro. Cancers.

